# Clinical outcome after endoscopic submucosal dissection for early gastric cancer of absolute and expanded indication

**DOI:** 10.1097/MD.0000000000006710

**Published:** 2017-04-28

**Authors:** Ju Seok Kim, Sun Hyung Kang, Hee Seok Moon, Eaum Seok Lee, Seok Hyun Kim, Jae Kyu Sung, Byung Seok Lee, Hyun Yong Jeong

**Affiliations:** Departments of Internal Medicine, Chungnam National University School of Medicine, Daejeon, Korea.

**Keywords:** early gastric cancer, endoscopic submucosal dissection, expanded indication

## Abstract

This study evaluated the clinical outcome of endoscopic submucosal dissection (ESD) in minute submucosa (SM) invasion or undifferentiated early gastric cancer (EGC) and analyzed factors related to local recurrence after ESD.

We retrospectively reviewed the chart of EGC patients, who underwent ESD at our tertiary hospital between January 2009 and 2015. The patients’ characteristics and clinical outcomes were compared among an absolute indication, minute SM invasion, and undifferentiated EGC group.

Of 885 total patients, 729 composed the absolute indication group; 65, the differentiated, SM invasion group; and 51, the undifferentiated, confined mucosa group. Follow-up was conducted for average (± standard deviation) 34.12 (± 10.6) months; as compared to the absolute indication group, both *en bloc* resection and curative resection rate were low in the other 2 groups, but there were no significant differences in procedure-related complication, local recurrence, and survival rate. Comparing the cases of ESD performed at our hospital from 2005 to 2009 with those performed between 2009 and 2015, *en bloc* resection (80.5% vs 89.1%, *P* = .001) and curative resection rate (86.2% vs 92.1%, *P* = .011) were higher in the latter study. Noncurative resection and tumor size of more than 2 cm were factors associated with local recurrence.

ESD in minute SM invasion or undifferentiated EGC showed an unfavorable short-term outcome as compared to that in the absolute indication group, but there were no differences in local recurrence and overall survival rate. Therefore, in minute SM invasion or undifferentiated EGC patients, ESD could be recommended as a therapeutic option.

## 1 Introduction

Early gastric cancer (EGC) was defined as a condition in which a lesion is limited to the mucosa or submucosa (SM), regardless of lymph node (LN) metastasis.^[1]^ In the past, surgical resection was the standard method of treatment; however, currently, endoscopic resection has been accepted as the primary treatment option for EGC in many countries.^[2,3]^ According to the recent guidelines, the absolute indication for endoscopic resection is differentiated adenocarcinoma, elevated lesions less than 2 cm and depressed lesions ≤ 1 cm without ulceration.^[4]^ However, these criteria are too stringent, thereby leading to more surgeries.^[5]^ Endoscopic submucosal dissection (ESD) can be used for the *en bloc* resection of even a lesion larger than 2 cm or accompanied by an ulcer, which is difficult to be treated by the existing endoscopic mucosal resection (EMR) and has fewer limitations due to the shape or position of the lesion.^[5,6]^ In addition, with the development of endoscopic instruments and improvement of operators’ techniques, the indications for endoscopic resection are expanding.^[7,8]^

Gotoda^[5]^ defined as the following criteria for the indication of endoscopic treatment of EGC: (1) regardless of the size, differentiated confined mucosal cancer without ulcer; (2) differentiated mucosal cancer with ulcer and diameter ≤ 30 mm; and (3) differentiated minute submucosal invasion cancer of diameter ≤ 30 mm. In addition, according to Hirasawa et al,^[9]^ undifferentiated mucosal cancer without ulcer and diameter ≤ 20 mm has a less possibility of LN metastasis. However, as a high possibility of LN metastasis or local recurrence in expanded indication, as compared to the existing absolute indication, has been shown, there remains a controversy about its safety, due to lack of large-scale research on it.^[10,11]^ In addition, tumor size, histology, invasion depth, and curative resection are known factors associated with the local recurrence after endoscopic resection, but they do not show consistent results in several studies.^[12,13]^

Thus, this study aimed to compare the clinical outcome and safety of ESD in the treatment of an absolute indication group and a minute SM invasion or undifferentiated group of EGC patients and find out the factors related to local recurrence after endoscopic resection.

## 2 Methods

### 2.1 Patients

A retrospective chart review was performed on patients who were histologically diagnosed with EGC at the Chungnam National University Hospital (Daejeon, Korea) and underwent ESD between January 2009 and January 2015. This study included patients aged over 18, who received treatment at our hospital and over 1-year follow-up. They were histologically classified into differentiated (well and moderately differentiated or mucinous adenocarcinoma, papillary) or undifferentiated (poorly differentiated adenocarcinoma, poorly cohesive or signet ring cell carcinoma) type. In addition, by invasion depth, they were classified into a confined mucosa or minute SM invasion group (<500 μm from the muscularis mucosa). Lastly, all patients were classified into the following 3 groups and analyzed: (1) absolute indication; (2) differentiated, SM invasion; and (3) undifferentiated, confined mucosa group. In all patients, computed tomography (CT) was performed to exclude any LN or distant metastasis. This study was approved by the Institutional Review Board of Chungnam National University Hospital.

### 2.2 ESD procedure

Patients were sedated with midazolam (Roche Korea), and their cardio-respiratory function was consistently monitored, whereas propofol was being added according to the patient's sedation. All ESDs were performed by 4 specialized gastrointestinal (GI) endoscopists (SHK, HSM, JKS, and HYJ). EGC was evaluated by using white-light endoscopy and chromoendoscopy with indigo-carmine solution and when necessary magnifying endoscopy with narrow-band imaging was also conducted. The EGC lesions were marked with argon plasma coagulation. In addition, epinephrine and indigo carmine containing the normal saline solution were injected. An ESD was performed by using an insulation-tipped diathermy (IT) knife or IT knife-2 (Olympus Medical, Japan) and high frequency generators (ICC200 or VIO 300D; ERBE Elekromedizin, Germany).

### 2.3 Pathological examination

The resected tissue specimens were spread wide, and fixed with a pin on a polystyrene plate, in 10% formalin. After the fixation, the specimens were grossly examined, and serial sectioning was performed, followed by histological mapping. The cellular tissue that accounts for over 50% of the entire tumor cell was classified into the main tissue form. All the resected samples were reviewed by our hospital GI special pathologists.

### 2.4 Definition

The definition of *en bloc* resection was a lesion resected by 1 piece. Curative resection was defined as *en bloc* resected tumors and lateral margin > 2 mm and basal margins > 0.5 mm without lymph-vascular invasion. In the case of piecemeal resection, the curative resection was defined as the presence of sufficient lateral and basal margins after reconstruction of tissue. Bleeding requiring endoscopic hemostatic procedure without any clinical symptom or laboratory abnormality was defined as minor bleeding, whereas significant bleeding involved symptoms such as melena or hematemesis or fall of hemoglobin level (>2 g/dL). Perforation was defined as a complication observed during the ESD procedure when free air was detected on chest or abdominal radiographic image. Gross types of lesions were classified by Japanese Gastric Cancer Association.^[4]^ Recurrent cancer at the resection site after 12 months was defined as local recurrence.

### 2.5 Follow-up

Complete blood cell count and radiography were performed a day after the implementation of ESD, and after confirming that there were no complications, a dietary treatment began. Scheduled endoscopy was performed 3, 6, and 12 months after the procedure, and annually thereafter, whereas biopsy was performed for all lesions with suspicious abnormality. At an interval of 6 months, chest radiography and abdominal CT were performed, and the implementation interval was controlled by the judgment of the physician.

### 2.6 Statistical analysis

The chi-square test and Fisher's exact test were used to compare the categorical variables, including patients’ characteristics, endoscopic outcomes, and clinical outcomes. Overall survival and disease-specific, recurrence-free rate were analyzed by the Kaplan–Meier method and the log-rank test. Odds ratios (OR) of the risk factor of local recurrence were analyzed by the logistic regression model. For the multivariate analysis, some variables, such as curative resection, tumor size, differentiation, and depth of invasion were adjusted. *P*-value was 2-sided, and less than .05 was considered significant. All statistical analyses were conducted using the SPSS version 18.0 (SPSS Inc., Chicago, IL).

## 3 Results

### 3.1 Patient and clinical characteristics

Excluding patients with beyond the expanded indication (n = 63) and follow-up loss (n = 11), 845 patients were enrolled, who were diagnosed with EGC and received ESD at our hospital and were under observation for progress. The mean (± standard deviation, SD) age of the patients was 64.7 (± 8.6) years and 72.8% (n = 615) were males. They were divided into the absolute indication (n = 729), differentiated, SM invasion (n = 65) or undifferentiated, confined mucosa group (n = 51). There was no statistical significant difference in the procedural outcomes of 4 endoscopists who performed ESD. In total, 845 patients were divided into 3 groups and compared (Table 1). As compared to the absolute indication group, there were no differences in gender and macroscopic type, but the tumor size was larger in the other 2 groups, and there was a significant difference in tumor location. In addition, the age of the undifferentiated, confined mucosa group was lower than that of the absolute indication group (*P* = .000). Of the total 845 patients, pre-ESD pathology of the differentiated and undifferentiated types was found in 808 and 37 patients, respectively, and post-ESD pathology was observed in 794 and 51 patients, respectively. The histological discrepancy from the differentiated type to undifferentiated type was observed in 22 patients and the reverse in 8. The histological discrepancy rate was 3.6% (n = 30). ESD was performed in 42 patients due to synchronous or metachronous EGC.

**Table 1:**
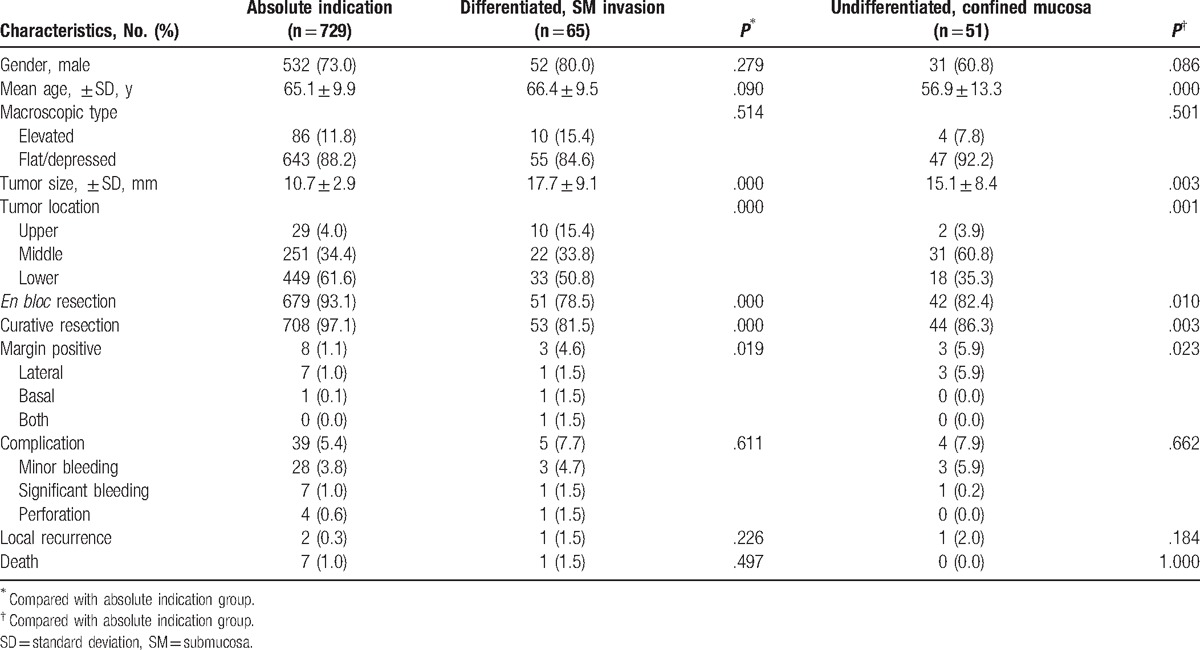
Patient characteristics and endoscopic outcomes in the absolute indication and differentiated, SM invasion and undifferentiated, confined mucosa groups.

### 3.2 Short-term outcomes of ESD

In all patients, the overall *en bloc* resection rate was 91.3%, and further dividing them into 3 groups, the rate was 93.1%, 78.5%, and 82.4%, respectively, in the absolute indication, differentiated, SM invasion and undifferentiated, confined mucosa group. As compared to the absolute indication group, significantly lower *en bloc* resection, and curative resection rate were observed in the other 2 groups. Of all patients, bleeding or perforation occurred in 5.7% (n = 48), and there were no differences in the differentiated, SM invasion group (*P* = .611) and undifferentiated, confined mucosa group (*P* = .662) as compared to the absolute indication group. Local recurrence occurred in 4 patients (0.4%); however, there was no significant difference among the 3 groups. During the same period, a total of 8 patients (0.8%) died, but there was no statistical significant difference in mortality among the 3 groups.

### 3.3 Long-term outcomes of ESD

**Figure 1:**
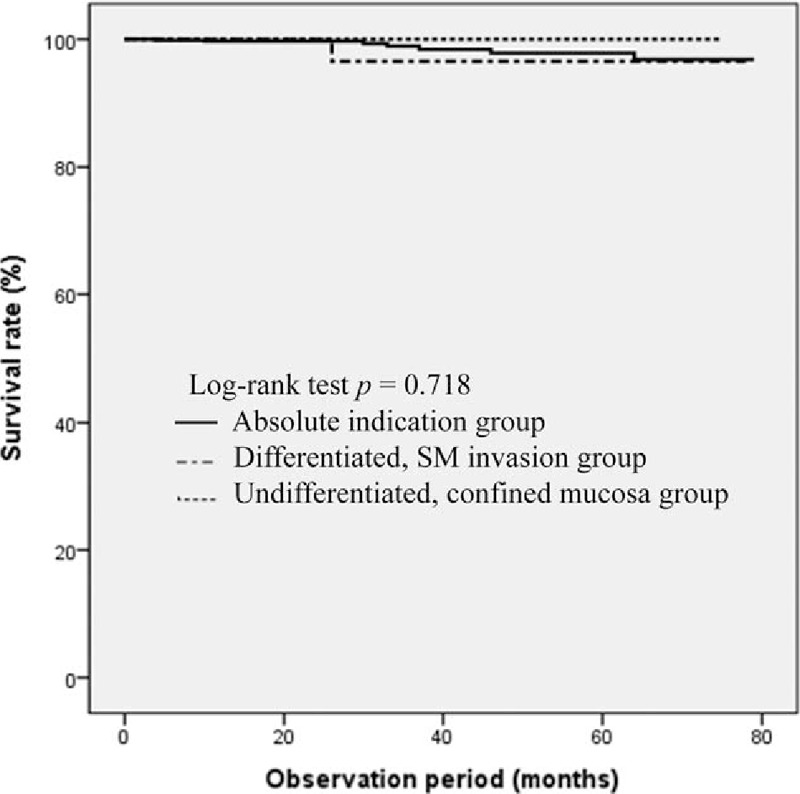
Kaplan–Meier estimates of overall survival rate.

**Figure 2:**
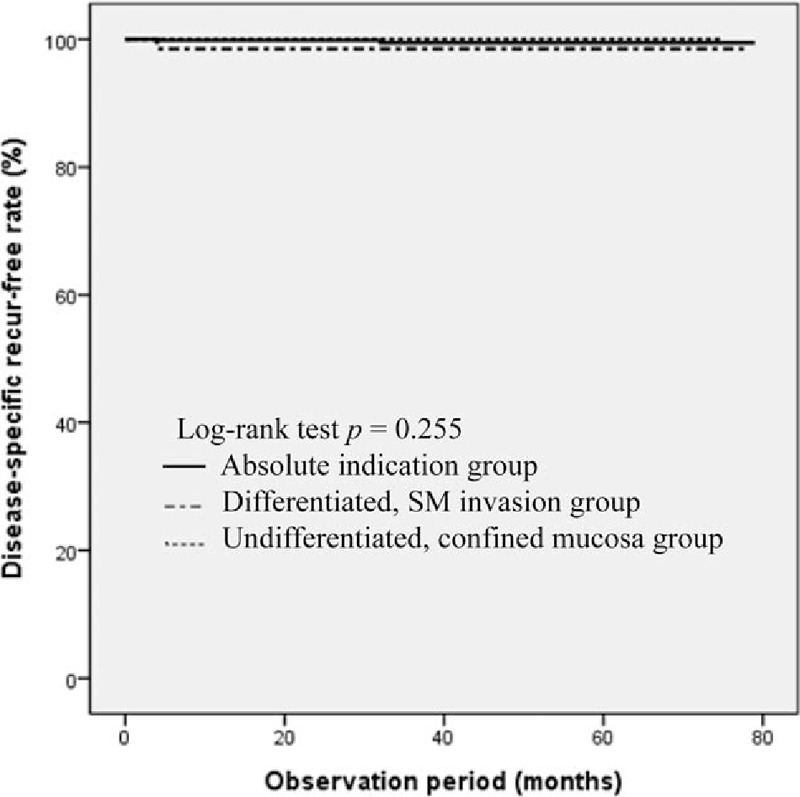
Kaplan–Meier estimates of disease-specific, recurrence-free rate.

The patients were divided into absolute indication (n = 729), differentiated, SM invasion (n = 65) and undifferentiated, confined mucosa (n = 51) group and their long-term outcomes of ESD were compared. During follow-up for average (± SD) 34.12 (± 10.6) months, 8 patients died, and the 5-year survival rates were 96.8%, 96.6%, and 100% in the absolute indication, differentiated, SM invasion and undifferentiated, confined mucosa group, respectively (Fig. 1). The result of a log-rank test showed that there was no difference in the overall survival rate among the 3 groups (*P* = .718). The 5-year disease-specific, recurrence-free rate was 99.5%, 98.5%, and 97.4% in the absolute indication, differentiated, SM invasion and undifferentiated, confined mucosa group, respectively (Fig. 2). There was also no difference in the disease-specific, recurrence-free rate among the 3 groups (*P* = .255).

### 3.4 Clinical outcome of ESD, according to the procedure period

The data analysis of the outcome of treatment in 210 EGC patients with ESD at our hospital from 2005 to 2009 was compared with the outcome of the study conducted between 2009 and 2015 (Table 2). To compare the studies, ESD was performed by the same endoscopists (HSM, JKS, and HYJ), except 1 (SHK). In the previous study, patients with beyond the expanded indication were included; hence, a total of 908 patients, including 63 patients who had been excluded from this study were analyzed. There were no differences in gender, age, depth of invasion, and differentiation between the 2 groups. However, *en bloc* and curative resection have significantly improved from 2009 onwards.

**Table 2:**
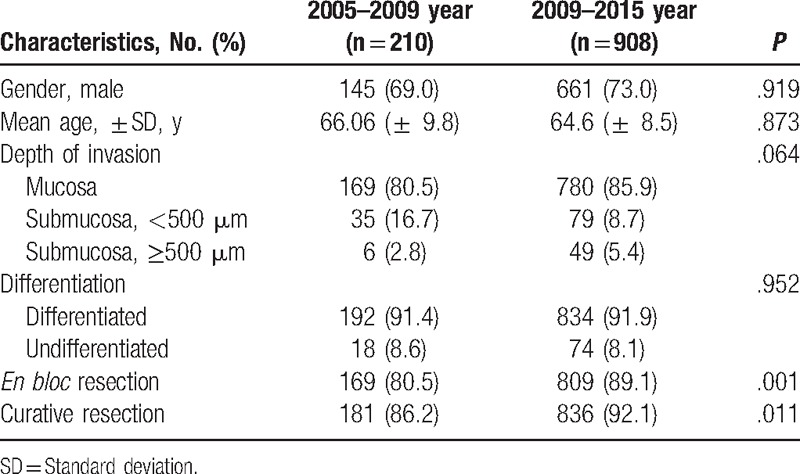
Clinical outcome of the endoscopic submucosal dissection, according to the procedure period.

### 3.5 Risk factor of local recurrence

In order to find out the risk factors of the local recurrence, a logistic analysis was conducted by adjusting the curative resection, tumor size, differentiation, and depth of invasion (Table 3). As a result, non-curative resection (OR 5.098, 95% confidence interval [CI] 1.059–24.544) and tumor size more than 2 cm (OR 7.487, 95% CI 1.696–33.064) were found to be independent risk factors of local recurrence. However, the degree of differentiation and depth of invasion were not associated with local recurrence.

**Table 3:**
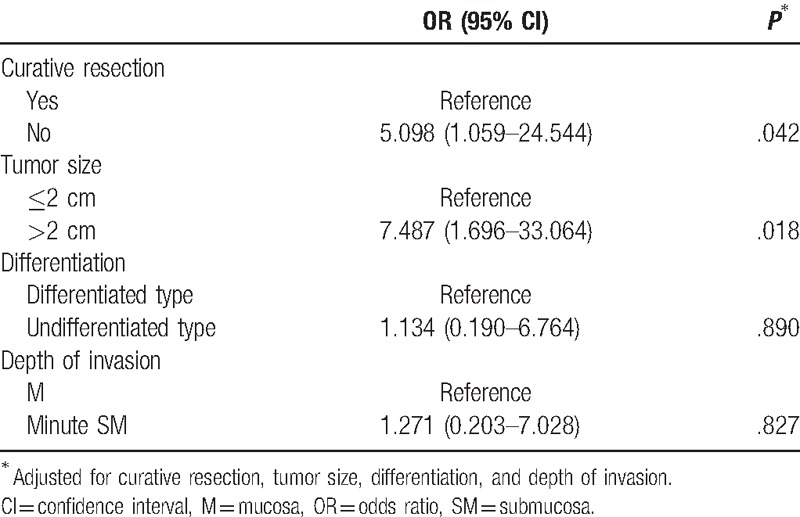
Multivariate analysis of risk factor of local recurrence after endoscopic submucosal dissection.

## 4 Discussion

According to the results of this study, as compared to the absolute indication group, the differentiated, SM invasion and undifferentiated, confined mucosa groups had lower *en bloc* and curative resection rates; however, there were no differences in complication, local recurrence, and overall survival rate after ESD among the groups. In addition, non-curative resection and tumor size more than 2 cm were factors related to local recurrence after ESD.

In EGC treatment, ESD preserves the normal anatomical structures of the stomach, does not cause functional disorders after surgery, and has a merit that it can reduce morbidity and mortality after surgery; thus, its indication is expanding.^[14]^ However, an important factor of endoscopic treatment is LN metastasis.^[15]^ Especially, there are studies showing that LN metastasis increases in undifferentiated or SM invasion EGC; thus, more attention is required.^[13,16]^ Kang et al^[17]^ reported that LN metastasis was 15.0% in SM invasion cancer (SM1) of ≤3 cm in size. Another study reported that LN metastasis was 1.7% in SM invasion cancer (SM1) < 2 cm.^[12]^ Undifferentiated EGC has 4.2% to 4.9% of LN metastasis in the mucosal invasion tumors and 19.0% to 23.8% of LN metastasis in SM invasion tumors.^[9,18]^ However, there are results of other studies, which are contrasting to this. LN metastasis was absent in SM invasion cancers ≤30 mm in size.^[19]^ According to a meta-analysis, as compared to the absolute indication, in the expanded indication, *en bloc* resection rate (93.6% vs 97.0%, *P* < .001), complete resection rate (87.8% vs 95.8%, *P* < .001), and curative resection rate (82.4% vs 94.0%, *P* < .001) were significantly lower, but there were no significant differences in morbidity (*P* = .22) and overall mortality (*P* = .37) between the 2 groups.^[20]^ This study, also, showed similar results, and as compared to the absolute indication group, although curative resection rate was low in the differentiated, SM invasion and the undifferentiated, confined mucosa groups, there were no differences in local recurrence and survival rate, which are important factors to judge the treatment result. Lastly, minute SM invasion or undifferentiated EGC showed a favorable long-term outcome similar to the existing absolute indication.

Recently, with the development of the endoscopic instruments and operation devices, indication of ESD is gradually expanding.^[7,8]^ Especially, undifferentiated EGC shows a subepithelial lateral spreading or discontinuous pattern, and narrow band image with magnifying endoscopy or confocal laser endomicroscopy helps to establish the boundary of a lesion.^[21,22]^ However, a question arises from the extent of the impact these developments have on the actual treatment outcome. Thus, our hospital ESDs data were analyzed by the procedure period. The clinical outcome of treatment of patients who had received an ESD by the same endoscopists between 2005 and 2009 were compared with the outcomes in this study. To keep the same criteria for inclusion, patients with undifferentiated, SM invasion EGC were also included in the analysis. There were no differences in gender, depth of invasion mean age, and differentiation between the 2 groups, but *en bloc* and curative resection rate were higher in the group of patients who had recently received an ESD. Lastly, the development of endoscopic instruments and better technique improved the actual treatment outcomes, and it is expected that this result would lead to the expansion of indication of ESD, but additional studies are still needed.

In this study, the overall bleeding rate after ESD was 5.1%, and the perforation rate was 0.6%, which were results similar to those in previous studies.^[23,24]^ It is also reported that procedure-related complications increase in undifferentiated or SM invasion.^[25]^ In this study, there was no difference in the SM invasion (7.7% vs 5.4%, *P* = .611) and undifferentiated group (7.9% vs 5.4%, *P* = .662) compared with absolute indication group. Moreover, in other recent studies, when comparing absolute indication with expanded indication, there was no difference in the incidence rate of complications between the 2 groups.^[20]^ Likewise, it is judged that ESD in minute SM invasion or undifferentiated EGC is a sufficiently safe procedure showing a similar complication rate to that of the absolute indication group.

Generally, the known factors related to local recurrence after the ESD of EGC are, tumor size, histology, invasion depth, and curative resection.^[12,13]^ This study analyzed risk factors in patients with local recurrence and showed results similar to those of other studies. Risks of local recurrence were significantly higher in noncurative resection and tumor size more than 2 cm, but there was no correlation found with differentiation and depth of invasion. To determine the depth of invasion, endoscopic ultrasound (EUS) may be performed before ESD. However, according to the research till now, it is inaccurate to evaluate the depth of invasion of a tumor by EUS, and especially, it is reported that it is as low as about 59% to 76% in undifferentiated EGCs.^[26,27]^ In addition, the accuracy of EUS also differs depending on the invasion depth of the tumor, and since depth of invasion tends to be underestimated in this case, it requires attention, and, further study.^[28]^ Therefore, it is necessary to carefully observe the progress of patients in whom these risk factors are predicted, and an additional surgical treatment should be considered according to the pathological result after the treatment.

The limitations of this study are as follows: first, it was a single-center study, and there were relatively few patients enrolled in each subgroup, which might affect the result of the study. In addition, it had a retrospective design.

ESD in the minute SM invasion or undifferentiated, confined mucosal EGC had a lower curative resection rate as compared to absolute indication, but there were no significant differences in procedure-related complications, local recurrence, and overall survival rate. It is judged that this was because of the development of the endoscopic instruments and operation devices as proven in this study. Non-curative resection and tumor size more than 2 cm were the factors related to local recurrence after ESD. In conclusion, ESD in the minute SM invasion or undifferentiated, confined mucosal EGC is safe, showing a favorable long-term outcome; thus, the expansion of the indication of ESD is expected, but further multi-center, prospective studies are needed to confirm our data.
